# Microencapsulation with Different Starch-Based Polymers for Improving Oxidative Stability of Cold-Pressed Hickory (*Carya cathayensis* Sarg.) Oil

**DOI:** 10.3390/foods12050953

**Published:** 2023-02-23

**Authors:** Qing Li, Lu Wang, Meiyu Zheng, Hanyu Lu, Yinying Liu, Yangguang Wang, Shengmin Lu

**Affiliations:** 1 State Key Laboratory for Managing Biotic and Chemical Threats to the Quality and Safety of Agro-Products, Zhejiang Provincial Key Laboratory of Fruit and Vegetables Postharvest and Processing Technology Research, Ministry of Agriculture and Rural Affairs Key Laboratory of Post-Harvest Handling of Fruits, Institute of Food Science, Zhejiang Academy of Agricultural Sciences, Hangzhou 310021, China; 2 College of Food and Pharmacy, Zhejiang Ocean University, Zhoushan 316022, China

**Keywords:** cold-pressed hickory oil, microcapsules, β-cyclodextrin, porous starch, oxidative stability

## Abstract

Hickory (*Carya cathayensis* Sarg.) oil is a nutrient-dense edible woody oil, with its unsaturated fatty acids accounting for more than 90% of total ones, and liable to oxidation spoilage. To efficiently improve its stability and expand its application fields, the microencapsulation of cold-pressed hickory oil (CHO) by the molecular embedding method and freeze-drying technique was performed using malt dextrin (MD), hydroxylpropyl-β-cyclodextrin (HP-β-CD), β-cyclodextrin (β-CD), or porous starch (PS) as a wall material. Two wall materials and/or their CHO microcapsulates (CHOM) with higher encapsulation efficiencies (EE) were selected to carry out physical and chemical characterizations using laser particle size diffractometer, scanning electron microscopy, Fourier-transform infrared spectroscopy, X-ray diffraction, thermogravimetric analysis, derivative thermogravimetry, and oxidative stability tests. Results indicated β-CDCHOM and PSCHOM had significantly higher EE values (80.40% and 75.52%) than MDCHOM and HP-β-CDCHOM (39.36% and 48.32%). The particle sizes of the two microcapsules selected were both widely distributed with their spans being more than 1 µm and a certain degree of polydispersity. Microstructural and chemical characterizations indicated that β-CDCHOM had comparatively stable structure and good thermal stability compared with PSCHOM. Storage performances under light, oxygen, and temperature showed that β-CDCHOM was superior to PSCHOM, especially in terms of thermal and oxidative stability. This study demonstrates that β-CD embedding can be applied to improve the oxidative stability of vegetable oils such as hickory oil and act as a means of preparing functional supplementary material.

## 1. Introduction

As a nut high in oil, hickory (*Carya cathayensis* Sarg.) kernels can be processed into oil by extracting with supercritical CO_2_ fluid, cold-pressed, ultrasonic-assisted, and aqueous enzymatic methods. Different extraction methods have great influence on the extraction efficiency of oils and their contents of various bioactive components. Among them, the cold pressed method is a green, safe, and rapid one, which can effectively retain the nutrients in vegetable oils [1]. Hickory oil is a nutritious, edible oil, containing a large number of unsaturated fatty acids and trace amounts of squalene, β-sitosterol, vitamin E, etc. [2]. However, it is prone to rancidity and deterioration during storage and transportation due to oxygen, moisture, light, microbial, and other factors, seriously affecting its quality and nutritional value. The loss and consumption of physiological functions and active ingredients of hickory oil limit its shelf life, resulting in waste of oil resources and economic losses [3].

Microencapsulation is a protection technology which uses various wall materials to wrap solid, liquid, or gaseous substances, thus forming small particles. Moreover, the microcapsules can slowly release, instantaneously release, or permanently preserve particles on the basis of retaining the original characteristics of the core material [4]. This technology is nowadays widely used in the field of functional food materials, with which oil can be stored in a wall material to form a solid powder that is easier to handle and disperse, protect its bioactive compounds, improve its stability, and prolong the shelf life [5], and so does for entrapped cold-pressed hickory oil (CHO) to be easily mixed into functional foods to expand its utilization. To achieve this, vacuum freeze-drying is the most suitable encapsulation means due to its high retention of nutrients and flavorings [6]. At the same time, it is necessary to find the suitable wall material for encapsulating CHO and examine its protective effect to the oil.

As for starch-based wall materials, maltodextrin (MD) has the characteristics of low sweetness, odorlessness, good aqueous solubility, low moisture absorption, better thickening, and carrier stability, and is regarded as one of the good wall materials for food microcapsules [7]. However, the biggest problem of this wall material is its low emulsifying capacity [8]. β-cyclodetrin (β-CD) is a cyclic oligosaccharide consisting of seven α-D-glucopyranose units with a stable structural cavity that is hydrophobic on the inside and hydrophilic on the outside [9], and therefore is commonly used to encapsulate hydrophobic substances such as essential oils, lipids, and oils to prevent their volatilization or oxidation [10]. Hydroxylpropyl-β-cyclodextrin (HP-β-CD), one of the most widely used CD derivatives, can improve the water solubility of β-CD while maintaining its hydrophobic cavity where oil is adsorbed and protected. Porous starch (PS) is a modified starch with numerous nano or micron-sized pore structures from the surface to the center of the granule, featuring high porosity and specific surface area, strong adsorption, and high biocompatibility, and thus is very suitable as a carrier for liquid foods or sensitive substances to enhance their stability and expand their application areas [11]. By comparing the microencapsulation performances of these four different starch-based polymers, the effects of their different characteristics on the suitability of oil microcapsules and the differences after microencapsulation will be explored.

In this paper, four microcapsules of cold-pressed hickory oils (CHOMs) entrapped by different starch-based polymers were prepared using molecular encapsulation and freeze-drying methods. Two microcapsules with higher encapsulation efficiency (EE) were chosen for physical and chemical characterizations and stability studies. The morphologies of microcapsules were observed by scanning electron microscopy (SEM) and their encapsulations were verified by Fourier-transform infrared spectroscopy (FT-IR), X-ray diffraction (XRD), thermogravimetric analysis (TG), and derivative thermogravimetry (DTG). The oxidative stability of CHOMs was determined from four aspects of light, oxygen, storage temperature, and time. The advantages and disadvantages of the two CHOMs were compared.

## 2. Materials and Methods

### 2.1. Materials and Chemicals

Hickory nut was obtained from Lin’an District, Hangzhou, Zhejiang Province, China. MD (99%, Biological reagent), HP-β-CD (99%, Biological reagent), β-CD (99%, Biological reagent) and PS (99%, Biological reagent) were purchased from Shanghai Yuanye Biotechnology Co. Ltd. (Shanghai, China). Anhydrous ethanol and petroleum ether (Boiling range: 30–60 °C, Analytical reagent) were obtained from Shanghai Lingfeng Chemical Reagent Co. Ltd. (Shanghai, China). Peroxide value (POV) rapid detection kit was purchased from Nanjing Jiancheng Technology Co., Ltd. (Nanjing, China). Deionized water was used throughout the experiment.

### 2.2. Preparation of CHO

The crude hickory oil was extracted by an oil cold presser (DD85G, Komet company, Besigheim, Germany) with parameters set at rotating head D15 and rotating speed of 20 rpm. The oil was placed in a brown glass container sealed with tinfoil wrapper to avoid light and stored at room temperature for 3–5 d to allow full natural sedimentation, and then vacuum filtered in a Büchner funnel by two layers of palm oil filter paper clamped with one layer of ordinary filter paper to obtain clarified oil [12]. The fatty acid composition of the extracted oil was determined using GC-MS method and the sums of unsaturated fatty acids and saturated fatty acids were 90.93 ± 011% and 9.41 ± 0.31%, as reported before [12]. The finished oil was stored at 4 °C for later use.

### 2.3. Preparation of CHOMs

Two grams of MD, HP-β-CD, β-CD, or PS was, respectively, dissolved into 30 mL of deionized water in a water bath at 60 °C and stirred magnetically at 300 rpm to form wall solutions. The preparation took 1–4 min according to the wall materials’ dissolution rate in water and the solutions were put in a refrigerator at 4 °C to cool below room temperature. CHO (6 g) was dissolved into 5 mL anhydrous ethanol by a vortex oscillator (XH-C, Baita Xinbao Instrument Factory, Changzhou, China). The dissolved oil solution was added dropwise into the wall solution in a ratio of wall material solution to the core one at 6:1 (*v*/*v*), with magnetic stirring at 300 rpm and 60 °C for 4 h, and then the mixture was centrifuged at 4 °C and 7000 rpm for 10 min. The granule layer was collected and 1.5 g was used to determine its total solids content by calculating the percent of its constant weight to wet weight and the rest was vacuum filtered in a Büchner funnel with filter papers same as above, placed at −40 °C for 24 h, and freeze-dried at 0.011 mbar and −80 °C for 72 h. The prepared CHOM samples, namely, MDCHOM, HP-β-CDCHOM, β-CDCHOM, and PSCHOM, were protected from day light degradation in brown glass bottles and stored in a drying basin.

### 2.4. EE Analysis

For determination of the CHO amount on the surface of microcapsules, two grams of microcapsules were accurately weighed, and 20 mL of petroleum ether was added, then mixed with shaking at around 300 rpm using a vortex oscillator (XH-C, Baita Xinbao Instrument Factory, Changzhou, China) for 5 min. The mixture was filtered with filter papers as described above in a Büchner funnel using a recycling water vacuum pump (SHZ-D III, Shanghai Keshen Instrument Co., Ltd., Shanghai, China), and the residue was then washed with 10 mL of petroleum ether and filtrated again. The combined filtrate was used to recover the oil and petroleum ether, respectively, by the rotary evaporation method. The collected oil was dried to constant weight in a hot air-drying oven at 60 °C, and the final weight was recorded as the content of the surface oil.

For determination of the total CHO, two grams of microcapsules were accurately weighed and extracted by the Soxhlet extraction method, using petroleum ether as the extractant, in a water bath at 60 °C for 6 h [13]. Then, the oil was rotatively evaporated to remove petroleum ether and dried to constant weight as the total oil content. Based on the above quantitative determination, the EE was calculated following Equation (1) [14].


(1) $$\mathrm{EE}\left(\%\right)=\frac{\mathrm{Total}\;\mathrm{Oil}-\mathrm{Surface}\;\mathrm{Oil}}{\mathrm{Total}\;\mathrm{Oil}}\times 100$$

### 2.5. Characterization of CHOMs

#### 2.5.1. Particle Size Distribution Analysis

The particle size distributions of β-CDCHOM and PSCHOM were determined using a Mastersizer 2000 laser diffraction particle size analyzer (Malvern Instruments Ltd., Worcestershire, UK) based on wet dispersion method. Briefly, 0.5 g freeze-dried sample was suspended in 10 mL deionized water and sonicated for 30 s before determination. Procedure of determination was strictly followed to that provided by the supplier. Three measurements were performed for each sample and the span of the particle size distribution was calculated using Equation (2).

(2) $$Span=\frac{D_{90}-D_{10}}{D_{50}}$$

where *D*_90_, *D*_10_, and *D*_50_ are the volume diameter at 90%, 10%, and 50% of the cumulative size, respectively.

#### 2.5.2. SEM Analysis

The microstructures of wall materials and their microencapsulated CHO powders were observed using a Hitachi 8100 high-resolution cold field emission scanning electron microscope (Hitachi, Ltd., Tokyo, Japan). The samples were attached to the sample stage with conductive double-sided tape and coated with a thin layer of sputtered gold under vacuum, then imaged with the scanning electron microscope.

#### 2.5.3. FT-IR Spectroscopy

A Bruker VERTEX 70 FT-IR spectrometer (Bruker Ltd., Karlsruhe, Germany) was used to scan β-CD, PS, β-CDCHOM, and PSCHOM. All samples were mixed with solid KBr powder and the transmittances were recorded at wave numbers between 4000 cm^−1^ and 500 cm^−1^, with scans number 64 and a resolution of 4 cm^−1^.

#### 2.5.4. XRD Analysis

XRD patterns of β-CD, PS, β-CDCHOM, and PSCHOM powders were recorded with a PANalytical X’Pert PRO powder diffractometer (PANalytical Ltd., Almelo, Netherlands) using Cu Kα radiation (λ = 0.1541 nm). The working voltage was 40 kV and the working current was 40 mA. The patterns were collected with a 2θ range from 5° to 40°. The average crystallite sizes of the samples were calculated with the Scherrer equation.

#### 2.5.5. TG and DTG Analyses

β-CD, PS, β-CDCHOM, and PSCHOM powders (5–10 mg) were placed in an alumina crucible, respectively. The comprehensive thermal analysis was carried out by Netzsch STA449 C thermal analyzer (Netzsch Ltd., Serb, Germany). The heating temperature was from 30 °C to 425 °C, with the heating rate being 10°C/min and the N_2_ flow rate maintained at 35 mL/min.

### 2.6. Oxidation Stability

#### 2.6.1. Effect of Light on POV of Microcapsules

Twenty grams of β-CDCHOM, PSCHOM, or CHO were separately placed in brown glass containers and sealed with tinfoil wrappers. Under the environment of dark or light (constant illumination, 5000 Lux, 50 Hz), each group was stored at room temperature (25 °C) for 15 days [15,16]. Two grams of samples from each test group were taken out every 2 days, and POV of the samples were detected by the peroxide value rapid detection kit.

#### 2.6.2. Effect of Oxygen on POV of Microcapsules

Twenty grams of β-CDCHOM, PSCHOM, or CHO samples were weighed, respectively, and stored in vacuum (anaerobic) or open (aerobic) containers at room temperature (25 °C) for 15 days in dark [15,16]. POV were measured by the peroxide value rapid detection kit every 2 days in each experimental group.

#### 2.6.3. Effect of Temperature on POV of Microcapsules

Twenty grams of β-CDCHOM, PSCHOM, or CHO samples were separately placed in an airtight container and stored in dark at 4 °C, 25 °C, and 40 °C, respectively, for 15 days [15,16]. The temperatures reflect environments of practical applications of oils. POV were measured by the peroxide value rapid detection kit every 2 days in each experimental group.

#### 2.6.4. Effect of Storage Time on POV of Microcapsules

The Schaal oven method [17] was adopted and slightly modified. Twenty grams of β-CDCHOM, PSCHOM, or CHO samples in an airtight container were, respectively, placed in dark in an electric hot blast drying oven at 60 °C for 15 days. Two grams of samples from each test group were taken out every 2 days, and POV of the samples were detected by the peroxide value rapid detection kit.

### 2.7. Statistical Analysis

Each group of the experiment was repeated three times, and the results were expressed as mean ± standard deviation (SD). Microsoft Excel (Microsoft Inc., Redmond, WA, USA) and Origin 2021b (OriginLab Inc., Northampton, MA, USA) were used to process and plot the data. SPSS Statistics 25 (IBM Inc., Armonk, NY, USA) was used for statistical analysis and ANOVA was performed on the data. The average values of different treatments were compared by Tukey’s test (*p* < 0.05).

## 3. Results and Discussion

### 3.1. Encapsulation Efficiency of Microcapsules

Encapsulation efficiency is an important factor in evaluating the potential of a wall material to encapsulate the entrapped substance and can reflect its content in the matrix of wall material. Total solids contents in CHOMs were found in the same trend as that of EE values of four wall materials to CHO in an ascending order were MD (39.36%), HP-β-CD (48.32%), PS (75.52%), and β-CD (80.40%) (Table 1). MD was reported to have low emulsifying capacity and stability as well as non-hydrophobic nature [8], which may have influenced on the encapsulation efficiency of its corresponding oil capsule powder. In addition, the thickening property of MD is not beneficial for its encapsulating CHO while HP-β-CD has high aqueous solubility but remains the hydrophobic cavity of β-CD, thus more compatible with dense core materials such as CHO. It was reported that PS has a natural advantage for entrapping flavor and oils due to its porous structure [18]. As a perfect wall material for embedding oil, β-CD is distinguished by its hydrophobic internal surface and hydrophilic external surface [19]. Since the EE values of MD and HP-β-CD to CHO were too low, more efficient microcapsules (i.e., β-CDCHOM and PSCHOM) were used for further analysis on their physical and chemical properties and oxidation stability.

### 3.2. Characterization of CHOMs

#### 3.2.1. Size Distribution of CHOMs

Particle size directly affects the appearance, fluidity, and dispersibility of microcapsule powder [20]. The particle size distributions of β-CDCHOM and PSCHOM were shown in Table 2. The sizes of both microcapsules showed a normally clear single-peaked distribution. β-CDCHOM had a *D*_10_ of 4.268 µm, *D*_50_ of 9.147 µm, and *D*_90_ of 17.335 µm, with a particle size span of 1.429 µm, while PSCHOM had a *D*_10_ of 4.979 µm, *D*_50_ of 9.380 µm, and *D*_90_ of 16.425 µm, with the particle size span of 1.220 µm. The difference of particle size span between the two microcapsules was small, but their span values were both more than 1 µm, indicating that their particle sizes were widely distributed and both had a certain degree of polydispersity [21].

#### 3.2.2. Microstructures of CHOMs

**Figure 1:**
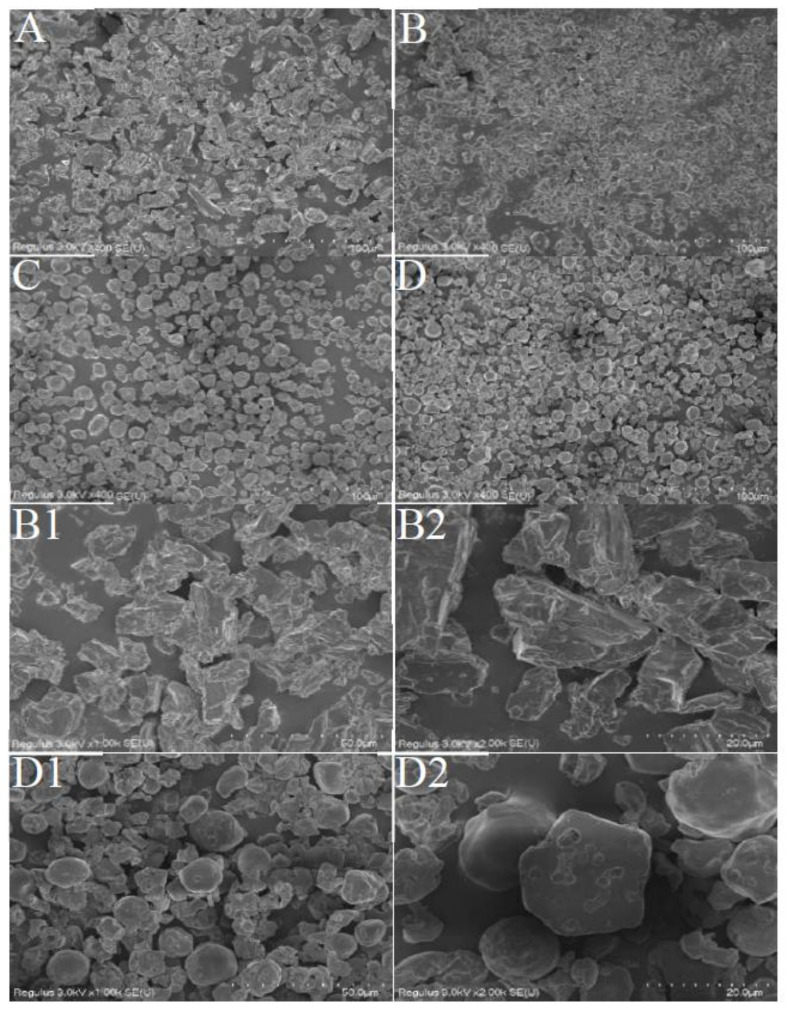
SEM of β-CD, PS and their CHO microcapsules. (**A**), β-CD (400-fold); (**B**), β-CDCHOM (400-fold); (**B1**), β-CDCHOM (1000-fold); (**B2**), β-CDCHOM (2000-fold); (**C**), PS (400-fold); (**D**), PSCHOM (400-fold); (**D1**), PSCHOM (1000-fold); (**D2**), PSCHOM (2000-fold). β-CD, β-Cyclodextrin; PS, Porous starch; CHO, cold-pressed hickory oil; β-CDCHOM, β-cyclodextrin cold-pressed hickory oil microcapsule; PSCHOM, Porous starch cold-pressed hickory oil microcapsule.

The β-CD powder had massive and irregular flakes with uneven sizes, and there were many small particles attached to their surfaces (Figure 1A). The distribution of β-CDCHOM was uniform, in mostly irregular columnar and rhombic flakes (Figure 1B). The amplified diagrams (Figure 1(B1,B2)) showed that the surface of β-CDCHOM had a few holes due to the embedding of CHO through freeze-drying, basically in a state of no cracks and large bulk. The results showed that β-CDCHOM was in new crystals formed by wall material and CHO, and had different shapes in layered, cylindrical, or spherical forms [22]. The PS powder was in an irregular circular structure with uniform dispersion and size, and its surface was filled with many holes (Figure 1C). The distribution of PSCHOM was relatively uniform and most of them were in irregular circular structures (Figure 1D). Compared with PS, PSCHOM had a blurred pore structure due to the inclusion of CHO. At the same time, the freeze-drying process led to the increase in pores and collapse of PSCHOM (Figure 1(D1,D2)) [23]. In addition, the aggregation of PS granules was not observed during the microencapsulating process, indicating that CHO did not deposit on surface of the granules, but entered their interiors through diffusion or infiltration [24]. However, it could also be seen that there were many ruptures in the structure of microcapsules, which might be due to the loss caused by the fluctuation of temperature during the preparation of microcapsules [19].

#### 3.2.3. FT-IR Spectra

**Figure 2:**
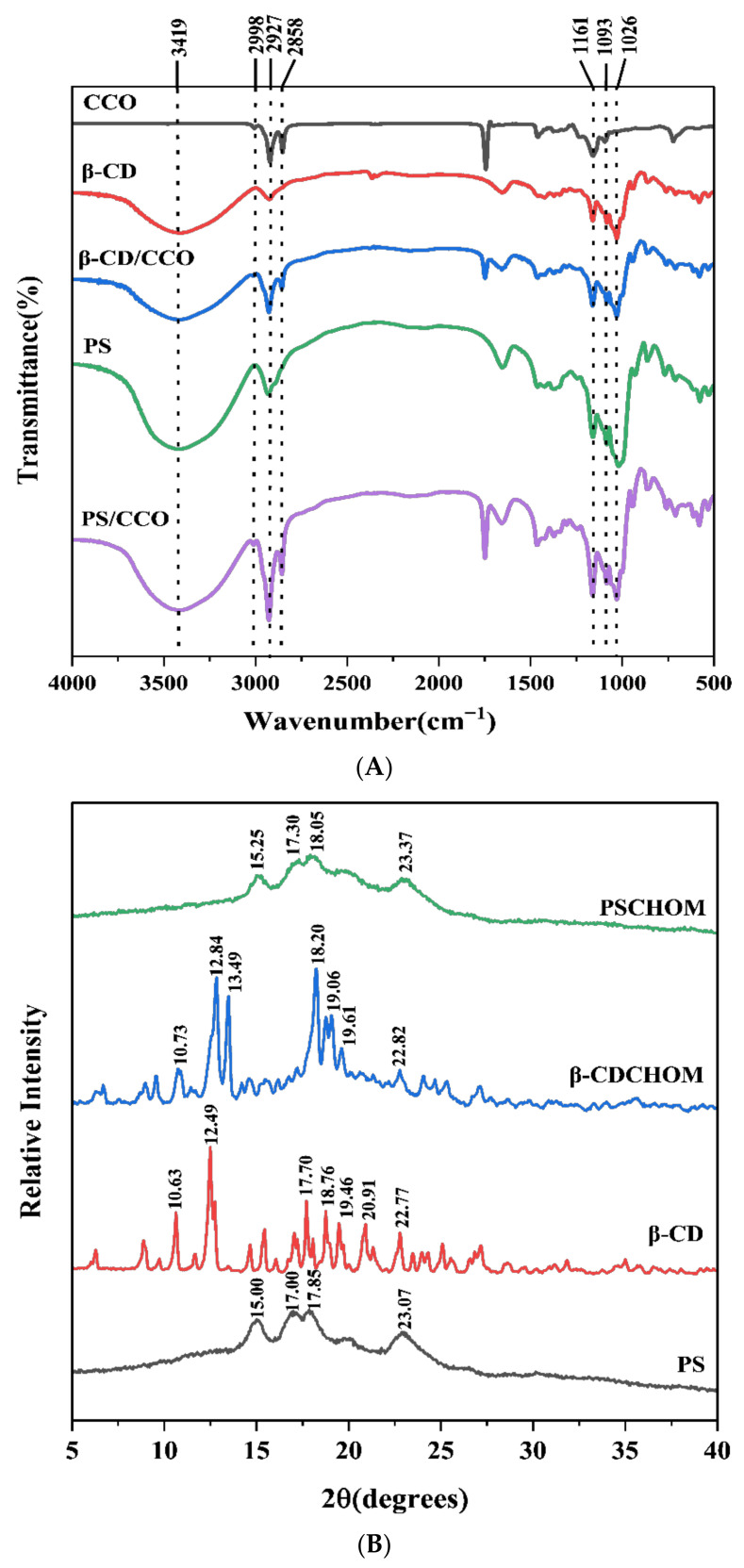
FT-IR spectra (**A**) and XRD spectrograms (**B**) of β-CD, PS and their CHO microcapsules. β-CD, β-Cyclodextrin; PS, Porous starch; CHO, cold-pressed hickory oil; β-CDCHOM, β-cyclodextrin cold-pressed hickory oil microcapsule; PSCHOM, Porous starch cold-pressed hickory oil microcapsule.

The molecular structures and chemical bonds of different compounds can be disclosed from their FT-IR spectra. Samples of β-CD, β-CDCHOM, PS, and PSCHOM had strong and wide absorption peaks at 3600~3000 cm^−1^ (Figure 2A). CHO, β-CD, β-CDCHOM, PS and PSCHOM had strong stretching vibration peaks at 2927 cm^−1^, indicating that these five constituents contained more saturated C-H bonds [25]. However, a strong stretching vibration peak representing the C=C bond was found in 3100~2700 cm^−1^ since CHO contained more oleic acid and linoleic acid which had a high degree of unsaturated bonds [26]. This characteristic peak also appeared in FT-IR spectra of β-CDCHOM and PSCHOM, indicating that CHO had been well embedded in these two wall materials. In the vicinity of 1747 cm^−1^, CHO, β-CDCHOM, and PSCHOM showed obvious C=C characteristic peaks [27] and they all showed distinct C-O and C-C characteristic peaks at 1161~1026 cm^−1^, indicating that CHO has been embedded into both starch-based microcapsules [28]. However, the overall stretching vibration intensity of β-CDCHOM was the weakest compared with those of CHO and PSCHOM, which was related to its stable cavity structure characteristics [29] and it was indicated that β-CDCHOM possessed the most stable encapsulation structure.

X-ray diffraction is usually used to study the relationship between the three-dimensional structural geometric properties and molecular properties of crystal materials. Due to the differences in the structures and molecular properties of β-CD and PS, the XRD spectra of β-CDCHOM and PSCHOM were also different (Figure 2B). β-CD had obvious diffraction peaks at 10.36°, 12.49°, 17.70°, 18.76°, and 20.91° while β-CDCHOM had strong diffraction peaks at 12.84°, 13.49°, 18.20°, 19.06°, and 19.61° at 2θ, i.e., they were both crystalline particles with regular and shaped structures and had a relatively complete crystal morphology [30]. Compared to β-CD, β-CDCHOM had an obvious different diffraction pattern with some peaks disappeared and others displaced in some degrees. The weakening or displacement, as well as the strengthening of some peaks indicated that the crystal structure of β-CD molecules had been changed after microencapsulation, and new crystal phases were generated after β-CDCHOM was formed. PS had obvious diffraction peaks at 2θ of 15.00°, 17.00°, 17.85°, and 23.07° while PSCDCHOM had those at 2θ of 15.25°, 17.30°, 18.05°, and 23.37°. Different from those of β-CD and β-CDCHOM, the diffraction peaks of PS and PSCHOM showed to be wider and more gentle, with overall spectra dispersed without exhibiting sharp Bragg peaks [31]. Compared with those of PS, the diffraction peaks of PSCHOM shifted to the right as a whole and showed a weakening trend due to the existence of CHO molecules, which would affect the lattice constant of PS [32]. Although CHO had little effect on the crystal morphology of PS, it could be proved that there was a new crystal phase formed in PSCHOM.

#### 3.2.4. Thermogravimetric Analysis and Derivative Thermogravimetry Results

Thermogravimetric (TG) is a technique to record the variation of material mass with temperature by heating the sample at a constant rate in a specific gas and measuring the change in sample mass with temperature through the internal precision balance of the instrument. It is mainly used to analyze the degradation process of the sample and its thermal stability [33]. The TG curve represented the cumulative weight loss of the sample changed with temperature, and its shape usually had a transition zone and a tilt zone. The first-order temperature derivative of the TG (DTG) curve reflects the relationship between the weight loss rate and temperature. TG-DTG combination can be used to well analyze the weight variation with temperature [34].

**Figure 3:**
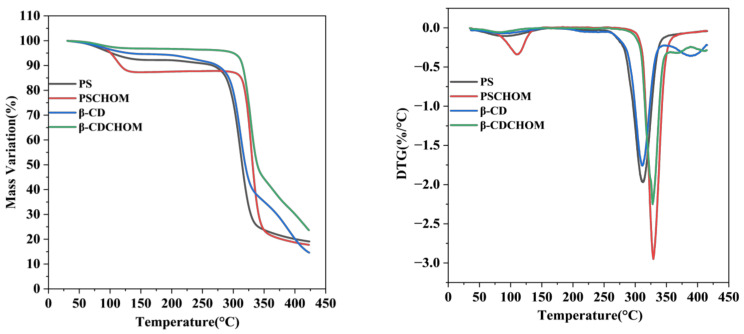
Integrated thermal analyses of β-CD, PS and their CHO microcapsules ((**left**): TG; (**right**): DTG). β-CD, β-Cyclodextrin; PS, Porous starch; CHO, cold-pressed hickory oil; β-CDCHOM, β-cyclodextrin cold-pressed hickory oil microcapsule; PSCHOM, Porous starch cold-pressed hickory oil microcapsule.

It was seen that the temperature dependent mass changes of β-CDCHOM and PSCHOM were mainly divided into three stages (Figure 3). At stage I (50–130 °C), the mass variation was slow, mainly for water evaporation, escape of volatile substances and some small molecules. Among the four samples, mass loss (11.3%) of PSCHOM was the most obvious because of the escape of some small molecules in CHO embedded in PS [35], and probably much free water in existence in the cavities of PS. Meanwhile, its weight loss rate of DTG was corresponding with the mass variation in TG, with the maximum at 109 °C. Contrarily, the mass loss of β-CDCHOM was only 3.7% due to its excellent protection towards small amounts of free water inside β-CD molecules [36]. At stage II (270–340 °C), the TG and DTG curves of the β-CDCHOM and PSCHOM all decreased sharply and the maximum mass decrease rates of β-CDCHOM (50.6%) and PSCHOM (62.8%) were found at 327.6 °C and 329.5 °C, respectively. At this stage, most of the wall materials began to decompose, and the chemical bonds in β-CD and PS molecules were cracked to produce gases such as CO_2_, H_2_O, and CH_4_, and the core material was also evaporated [37]. It was found that the changing trend was closely related to the structural characteristics and temperature sensitivity of the two starch granules. At stage III (350–425 °C), the mass decrease in microcapsules slowed down, mainly due to the complete carbonization of residual β-CDCHOM and PSCHOM, and the thermal decomposition was completed. The results of the three stages showed that the thermal stability of β-CDCHOM was better than that of PSCHOM. In summary, β-CDCHOM had comparatively stable structure and good thermal stability at temperatures less than 300 °C, which was suitable for food processing and application.

#### 3.2.5. Oxidation Stability Results

**Figure 4:**
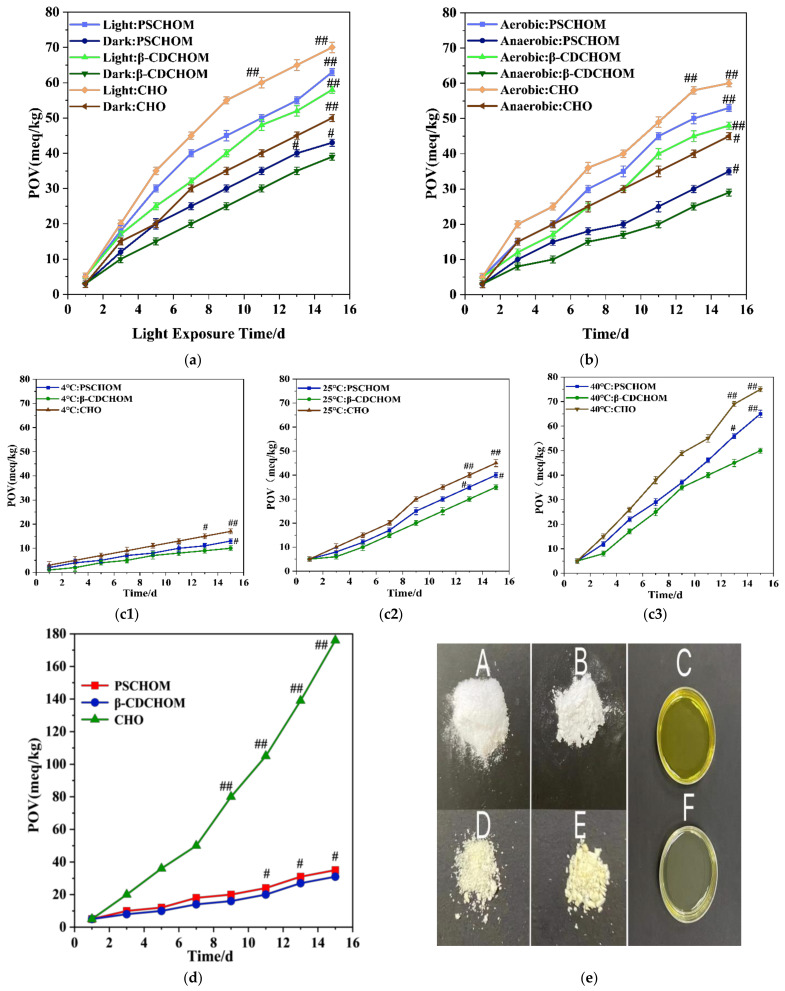
Effects of light (**a**), oxygen (**b**), temperature (c: (**c1**), 4 °C; (**c2**), 25 °C; (**c3**), 40 °C) and storage time at 60 °C protected from daylight and exposure from oxygen (**d**) on POV of CHO and its microcapsules during 15 days of storage, and appearance photos of CHO and its microcapsules newly prepared and after storage at 60 °C for 15 days (**e**). For photos, A, B and C refer to the newly prepared β-CDCHOM, PSCHOM, and CHO, respectively, and D, E, F stand for the corresponding samples after storage. Compared to #, ## represents significance at *p <* 0.05.

The POV of CHO and its microcapsules under light or dark increased in varying degrees (Figure 4a), with higher values found in light groups than dark ones. The CHO group under light had the highest POV, with its value increased by 65 meq/kg after 15 days of storage at ambient temperature, whereas β-CDCHOM in the dark had the least POV increase (40 meq/kg). Both microcapsules regardless of light and dark reduced the rise in POV of CHO entrapped, and the shielding protection by wall materials might delay and attenuate the self-oxidation and photooxidation rates of CHO, which could efficiently prolong its storage period. β-CDCHOM had superior performance in oxidative stability over PSCHOM under both light and dark storage conditions, probably because β-CD was composed of 7 glucose molecules, with a hydrophobic molecular cavity, which was easier to adsorb CHO and form stable supramolecular structures with CHO [38].

Figure 4b showed POV increases of CHO and its microcapsules under oxygen presence were significantly higher than those under anaerobic condition (*p <* 0.05). The POV of CHO, β-CDCHOM, and PSCHOM under oxygen presence increased by 55 meq/kg, 43 meq/kg, and 48 meq/kg, respectively, while those under anaerobic condition increased by 42 meq/kg, 26 meq/kg, and 36 meq/kg, respectively, after 15 days of storage at ambient temperature. Anaerobic environment significantly reduced rise in POV of CHO regardless of encapsulation, indicating CHO was sensitive to oxygen and susceptible to oxidation [3]. However, these two starch-based wall materials caused further reduction in POV increase in entrapped CHO under both aerobic and anaerobic conditions, probably because the wall materials could better isolate oxygen and block the entry of microorganisms to slow down the oxidation process of CHO. In addition, the superior performance of β-CDCHOM over that of PSCHOM under both aerobic and anaerobic conditions might be because hydrophobic molecular cavity of β-CD had more capacity to adsorb hydrophobic CHO than porous structure in PS [4,10], as shown in higher EE value for the former than the latter (Table 1). As a result, β-CD can isolate more oxygen and effectively slow down the oxidation rate of CHO in β-CDCHOM, which was more favorable for the storage of CHO.

The POV of CHO and its microcapsules increased obviously with the increase in storage temperature (Figure 4(c1–3)). From the data, low temperature storage efficiently inhibited the oxidation of CHO resulting in lower POV and the encapsulation had significant protective effects at *p <* 0.05. Therefore, the optimal storage temperature of CHO and its microcapsules was 4 °C. Moreover, the higher the temperature was, the more significantly the POV of the two microcapsules was lower than that of CHO (*p <* 0.05). The results indicated that the wall material structure of microcapsules could effectively protect CHO and reduce the influence of temperature on CHO. Overall, the performance of β-CDCHOM was better than that of PSCHOM under all three storage temperature, with the best protection at 40 °C. The possible reason is that β-CD is insensitive to temperature compared to PS, and the supramolecular structure of β-CD might provide more hydrogen bonds to connect with CHO [4,5], making the load of CHO to β-CD closer. Thus, β-CD can effectively reduce the impact of temperature changes on the embedded CHO, and is more suitable for CHO storage.

The POV value of CHO continued to increase during storage at 60 °C, protected from room light and isolated from oxygen, up to 176 meq/kg on the 15th day (Figure 4d). The overall increase in POV values of β-CDCHOM and PSCHOM was significantly lower than that of CHO, with the highest being 31 meq/kg and 50 meq/kg, respectively. This was because the external environmental factors such as light, temperature, and oxygen had less influence on CHO after microencapsulated by wall materials. In terms of β-CDCHOM and PSCHOM, the overall trend of POV increase in β-CDCHOM was lower than that in PSCHOM, indicating that the oxidation stability of β-CDCHOM was better than that of PSCHOM. Although there was little difference in POV values of β-CDCHOM and PSCHOM during day 1–5, the POV value of PSCHOM increased significantly, 1.4 times higher than that of β-CDCHOM on the 7th day, and it was continuously higher than that of β-CDCHOM thereafter. The possible reason was that PS was more sensitive to temperature changes because of its porous structure [11]. In addition, after 15 days of storage at 60 °C, the POV of CHO was 5.7 times that of β-CDCHOM. The CHO became transparent after storage for 15 days (Figure 4e(F)), and there was no color and fragrance of the crude oil (Figure 4e(C)), which might be caused by the oxidative discoloration of liposoluble pigments in CHO. Fortunately, microencapsulation could solve this problem. Primarily, the appearance of two microcapsule powders newly prepared were both milky white granular, without impurity or agglomeration phenomenon (Figure 4e(A,B)). After storage for 15 days, the color of two microcapsules was yellow and there was agglomeration (Figure 4e(D,E)), which might be caused by the oxidation of CHO in the microcapsules. β-CDCHOM had a small amount of small agglomeration (Figure 4e(D)), while PSCHOM was more serious in agglomeration (Figure 4e(E)), which also intuitionally confirmed that the oxidation stability of β-CDCHOM was better, and β-CD had a more stable microcapsule structure.

## 4. Conclusions

In this study, four starch-based (MD, HP-β-CD, β-CD, and PS) CHO microcapsules were prepared by molecular embedding method and freeze-drying technology. β-CD and PS, which had higher EE values on CHO, were selected for their CHO microcapsules’ characterization and oxidation stability tests. The particle size spans of the two microcapsules were both more than 1 µm and their difference was minimal. Microstructure observation, FT-IR spectra, XRD spectrogram, and TG-DTG combination analysis confirmed the encapsulation between the wall material and CHO and the results indicated that β-CDCHOM had comparatively stable structure and good thermal stability compared with PSCHOM, which was more suitable for food processing and production application. The storage stability tests showed that β-CDCHOM significantly improved the oxidative stability of CHO, which confirmed the effectiveness of encapsulation process. This study revealed that different starch-based polymers could be potentially used to improve the oxidative stability of vegetable oils.

## Figures and Tables

**Table 1:** Encapsulation efficiency of different starch-based wall materials to CHO.

Starch-Based Polymers	Total Solids Contents in CHOMs (%)	EE (%)
MD	29.02 ± 0.01 ^c^	39.36 ± 0.06 ^c^
HP-β-CD	30.13 ± 0.02 ^c^	48.32 ± 0.09 ^c^
β-CD	37.62 ± 0.01 ^a^	80.40 ± 1.02 ^a^
PS	34.87 ± 0.01 ^b^	75.52 ± 2.17 ^b^

MMD, Malt dextrin; HP-β-CD, Hydroxylpropyl-β-cyclodextrin; β-CD, β-Cyclodextrin; PS, Porous starch; CHOMs, cold-pressed hickory oil microcapsules. Different lowercase letters superscripted after data indicate significance at *p* < 0.05.

**Table 2:** Particle size distribution of cold-pressed hickory oil microcapsules.

Microcapsules	*D*_10_ (µm)	*D*_50_ (µm)	*D*_90_ (µm)	Span (µm)
β-CDCHOM	4.268	9.147	17.335	1.429
PSCHOM	4.979	9.380	16.425	1.220

β-CDCHOM, β-cyclodextrin cold-pressed hickory oil microcapsule; PSCHOM, Porous starch cold-pressed hickory oil microcapsule; *D*_10_, *D*_50_, and *D*_90_ are the volume diameter at 90%, 10%, and 50% of the cumulative size, respectively.

## Data Availability

The data presented in this study are available on request from the corresponding author. The data are not publicly available due to privacy.

## Abbreviations

CHO, Cold-pressed hickory oil; CHOM, Cold-pressed hickory oil microcapsules; MD, Maltodextrin; H-β-CD, Hydroxylpropyl-β-cyclodextrin; β-CD, β-cyclodextrin; PS, Porous starch; MDCHOM, Malt dextrin cold-pressed hickory oil microcapsules; H-β-CDCHOM, H-β-cyclodextrin cold-pressed hickory oil microcapsules; β-CDCHOM, β-cyclodextrin cold-pressed hickory oil microcapsules; PSCHOM, Porous starch cold-pressed hickory oil microcapsules; EE, Encapsulation efficiency; SEM, Scanning electron microscopy; FT-IR, Fourier-transform infrared spectroscopy; XRD, X-ray diffraction; TG, Thermogravimetric analysis; DTG, Derivative thermogravimetry; POV, Peroxide value.
